# Multiple Roles of Diatom-Derived Oxylipins within Marine Environments and Their Potential Biotechnological Applications

**DOI:** 10.3390/md18070342

**Published:** 2020-06-30

**Authors:** Nadia Ruocco, Luisa Albarano, Roberta Esposito, Valerio Zupo, Maria Costantini, Adrianna Ianora

**Affiliations:** 1 Department of Marine Biotechnology, Stazione Zoologica Anton Dohrn, Villa Comunale, 80121 Napoli, Italy; nadia.ruocco@gmail.com (N.R.); luisa.albarano@szn.it (L.A.); roberta.esposito@szn.it (R.E.); ianora@szn.it (A.I.); 2 Department of Biology, University of Naples Federico II, Complesso Universitario di Monte Sant’Angelo, Via Cinthia 21, 80126 Napoli, Italy

**Keywords:** biotechnology, chemical ecology, diatoms, oxylipins

## Abstract

The chemical ecology of marine diatoms has been the subject of several studies in the last decades, due to the discovery of oxylipins with multiple simultaneous functions including roles in chemical defence (antipredator, allelopathic and antibacterial compounds) and/or cell-to-cell signalling. Diatoms represent a fundamental compartment of marine ecosystems because they contribute to about 45% of global primary production even if they represent only 1% of the Earth’s photosynthetic biomass. The discovery that they produce several toxic metabolites deriving from the oxidation of polyunsaturated fatty acids, known as oxylipins, has changed our perspectives about secondary metabolites shaping plant–plant and plant–animal interactions in the oceans. More recently, their possible biotechnological potential has been evaluated, with promising results on their potential as anticancer compounds. Here, we focus on some recent findings in this field obtained in the last decade, investigating the role of diatom oxylipins in cell-to-cell communication and their negative impact on marine biota. Moreover, we also explore and discuss the possible biotechnological applications of diatom oxylipins.

## 1. Introduction

Diatoms are unicellular photosynthetic eukaryotes (class *Bacillariophyceae*), the primary production of which is the major driving force for incorporating organic carbon into the oceans thereby playing a central role in the biological carbon pump from the surface to deep sea [1,2]. Diatoms account for the highest number of plant species living in both marine and freshwater ecosystems and, due to a large variety of structural features, an estimation of the right number of species appears to be extremely difficult [3,4,5]. The chemical ecology of diatoms has been widely discussed, since the discovery of secondary metabolites, which negatively affect the reproduction of marine invertebrates. Known as the paradox of diatom–copepod interactions in pelagic food webs, since diatoms can both provide a source of energy for copepod larval growth and reduce their fecundity and/or hatching success [6,7,8].

This biological model is new and has no other equivalent in marine plant–herbivore systems, since most negative plant–animal interactions are generally related to repellent or poisoning processes, but never to reproductive failure (reviewed by Ianora and Miralto [9]). Moreover, when environmental conditions (sunlight intensity or nutrient concentrations) are optimal for the massive production of diatoms (algal blooms), the negative influence of toxic compounds may severely impact target consumers [10,11,12,13,14].

The enzymatic cascade leading to oxylipin production involves lipoxygenase (LOX)/hydroperoxide lyase (HPL) enzymes, which convert polyunsaturated fatty acids (PUFAs) into fatty acid hydroperoxides that are, in turn, converted into a plethora of compounds through mechanisms that are still largely unknown [15,16,17,18,19,20]. Oxylipins are a large family of compounds comprising polyunsaturated aldehydes (PUAs), firstly identified from *Thalassiosira rotula* [7], and other fatty acid derivatives with hydroxy-, keto-, oxo- and hydroxy-epoxy units, generically named non-volatile oxylipins [21] and recently reported as linear oxygenated fatty acids [22]. Several studies have suggested that, in addition to PUAs and oxygenated fatty acids, fatty acid hydroperoxides can trigger impacts on marine biota because these primary LOX products are reactive oxygen species (ROS), inducing DNA and protein damage that contribute to cell ageing [19]. Despite the negative impact observed in marine invertebrates, some volatile oxylipins were also proposed as odour compounds, attracting herbivores towards their food, thus suggesting that the function of oxylipins could change depending on the ecological context of diatom–invertebrate interactions [23,24,25].

Lipoxygenases (LOXs) constitute a family of dioxygenases that catalyse the oxygenation of free and esterified polyunsaturated fatty acids containing a (1Z,4Z)-penta-1,4-diene system to produce the corresponding hydroperoxy derivatives [25]. LOXs are expressed in plants [26] and in the animal kingdom [27,28], but have not been found in bacteria and yeast [29].

Chemical analyses of mono-algal cultures has revealed strictly LOX species-specificity [30], where the most common pathway shared by different genera of diatoms rely on a 15*S*-LOX activity, and a minor group of oxylipins are the products of 5-LOX, 8-LOX, 9*S*-LOX, 11-LOX, 12-LOX and 14-LOX [19,20,22,30,31,32,33,34] activity, depending on the specific regiochemistry of carbon oxidation. Oxylipin quantification and variation in time and space has been evaluated in field studies [35,36,37,38,39,40,41]. A recent survey demonstrated that oxylipin pathways in diatoms were mostly based on the oxygenation of hexadecatrienoic, eicosapentaenoic (EPA) and docosahexaenoic (DHA) acids, and, within phytoplankton communities, these secondary metabolites largely derived from diatoms [42]. Moreover, daily fluctuations of PUAs were more correlated to the cellular physiological state of diatoms than exclusively to the taxonomical composition of phytoplankton communities [43].

Interestingly, terrestrial plants also produce oxylipins in response to pathogen infections but differently from those described in marine diatoms. LOX substrates mostly consist of linoleic acid, α-Linolenic acid and hexadecatrienoic acid [44,45,46,47]. In analogy to plants, the production of oxylipins in diatoms was considered as a chemical defence against grazers. In fact, diatom-based diets or treatments with pure molecules induced a detrimental effect on gamete viability, embryogenesis and larval fitness of marine invertebrates, such as polychaetes, echinoderms, ascidians, crustaceans and molluscs [48].

Diatoms also produced a variety of bioactive secondary metabolites acting as chemical signals within phytoplankton communities [49,50,51,52]. Since PUAs were demonstrated to inhibit cell growth in diatom cultures and associated bacteria [53,54,55,56,57,58], a possible role as allelochemicals/infochemicals regulating the ecological success of diatom populations was proposed [59,60,61,62]. A few studies additionally demonstrated that PUAs exerted antiproliferative activities on human cancer cell lines promoting the activation of apoptotic pathways [7,63]. These findings have thus suggested that oxylipins could be used as a suitable source of new anticancer therapies.

## 2. Detrimental Impact of Oxylipins on Marine Invertebrates

**Figure 1:**
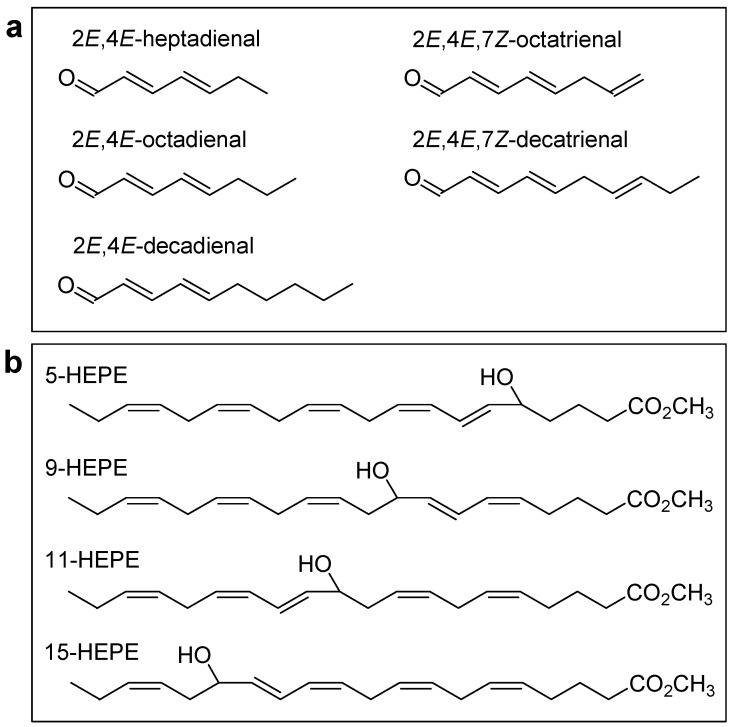
Chemical structures of commercially available PUAs (**a**) and HEPEs (**b**) used in experiments evaluating harmful effects of oxylipins on invertebrate reproduction and survival. Oxylipins were designed using ChemDraw Pro v8.0 software.

In the last decade, several studies have explored the negative impact of oxylipins, mainly using the pure molecules of commercially available PUAs, 2*E*,4*E*-heptadienal (HD), 2*E*,4*E*-octadienal (OD), 2*E*,4*E*,7*Z*-octatrienal (OT), 2*E*,4*E*-decadienal (DD) and 2*E*,4*E*,7*Z*-decatrienal (DT), and four oxygenated fatty acids with hydroxy functionalities, called hydroxyacids, 5-hydroxy-6*E*,8*Z*,11*Z*,14*Z*,17*Z*-eicosapentaenoic acid (5-HEPE), 9-hydroxy-5*Z*,7*E*,11*Z*,14*Z*,17*Z*-eicosapentaenoic acid (9-HEPE), 11-hydroxy-5*Z*,8*Z*,12*E*,14*Z*,17*Z*-eicosapentaenoic acid (11-HEPE) and 15-hydroxy-5*Z*,8*Z*,11*Z*,13*E*,17*Z*-eicosapentaenoic acid (15-HEPE). The chemical structures are reported in Figure 1.

All these studies were performed on the reproduction of sea urchins, tunicates and copepods, which are common diatom feeders in both benthic and planktonic environments.

**Figure 2:**
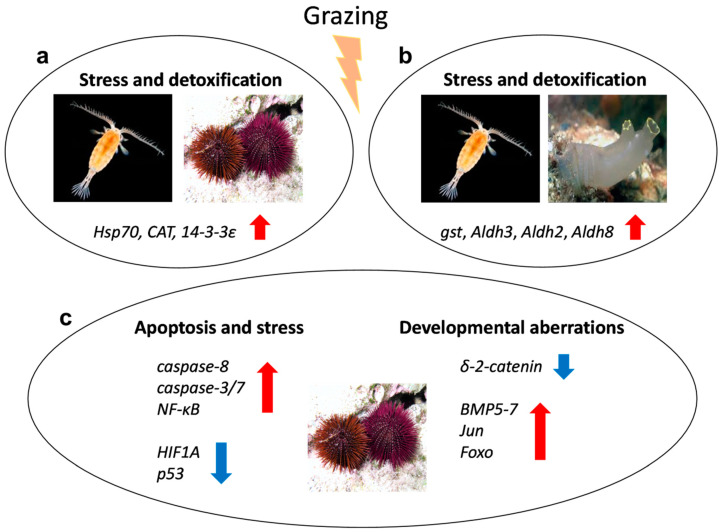
Molecular response of marine invertebrates to diatom’s oxylipins; possible common molecular pathway between copepods vs. sea urchins (**a**) and copepods vs. tunicates (**b**), together with some of the mostly strongly affected genes in sea urchins (**c**). Red arrows indicate upregulation of gene expression; blue arrows indicate downregulation of genes. Photos of copepods, sea urchins and tunicates were retrieved from the website https://www.marinespecies.org/.

The greatest novelty of these studies has been the introduction of genomic approaches to explain the effects of oxylipins on gene expression patterns in these organisms. Comparisons of these results indicate that some common pathways are activated in response to grazing of diatom-producing oxylipins by these marine invertebrates belonging to different phyla, as shown in Figure 2.

### 2.1. Sea Urchins

Overall, the experimental evaluation of the effects of oxylipins on sea urchins were mainly conducted through in vitro tests with the pure molecules of PUAs and HEPEs (Table 1).

Firstly, Romano et al. [64] demonstrated that, when the eggs of the sea urchin *Paracentrotus lividus* were incubated with four different PUAs (DD, OD, OT and HD), a severe dose-dependent block of the first cleavage was induced, with decadienal exerting the greatest activity. Moreover, at lower concentrations (1.32–5.26 μM), decadienal induced an increase in the number of malformed and delayed embryos at 48 h post-fertilisation (hpf, pluteus stage), with a high number of positive embryos to the Tdt-mediated dUTP nick end labelling (TUNEL) assay, indicating imminent death in embryos and larvae [64]. Subsequently, treatments with DD on sea urchin embryos exhibited increasing levels in the production of endogenous nitric oxide (NO) in a dose-dependent manner [65]. At high DD concentrations (>2.5 μg/mL), NO levels led to the activation of apoptotic events, whereas, at low concentrations (0.25 μg/mL), NO protected sea urchin embryos against teratogenesis by upregulating *hsp70* gene expression. However, at the pluteus stage, NO was not able to exert its positive role, since *hsp70* and *nitric oxide synthase* levels decreased and *caspase-8* gene, that normally initiates apoptotic signalling, was found upregulated [65]. The effects of two PUAs, HD and OD, together with the already studied DD, were investigated on embryo development of *P. lividus* [67]. The authors reported that PUAs caused similar malformations affecting the apex and the arms in a dose-dependent manner, with DD inducing the strongest effects, acting in a very narrow range of concentrations (0.5–2.5 μM) in comparison to HD and OD (1.0–6.0 and 2.0–9.0 μM, respectively). The same authors conducted post-recovery experiments revealing that treated embryos were able to recover depending on both PUA concentrations and washing time [67]. The same PUAs (DD, HD and OD) were also tested on embryo development of the Pacific sea urchin *Echinometra mathae* [69]. PUA toxicity scale (HD>OD>DD), evaluated at the *morula* and *pluteus* stages, was in contrast with previous results [67] and explained as an inverse correlation between PUA toxicity and carbon chain length. Specifically, the authors showed that HD induced 97% of abnormal plutei, while OD and DD induced lower percentages (72% and 28%, respectively) at 0.125 mg/mL. This result revealed a possible species-specific sensitivity, rendering sea urchins of different genera and/or living in different habitats differentially responsive to environmental toxins [69].

As mentioned above, several studies were also conducted on HEPEs, another class of oxylipins belonging to the non-volatile oxygenated fatty acids. Varrella et al. [72] tested the impact of 5- and 15-HEPEs on the sea urchin *P. lividus* for the first time. Experimental data showed that both HEPEs were not able to block the first cleavage, and, compared to the effects already observed for PUAs [67], HEPEs were found to be less active even if they induced the same types of malformations in embryos and larvae. However, HEPEs caused a developmental delay still detectable at a concentration of 7 μM, which prevailed at 30 μM, where treated embryos were all at the early pluteus stage instead of pluteus stage [72]. Conversely to PUAs [67], post-recovery experiments indicated that embryos were unable to undergo normal development when eggs were washed in seawater without HEPEs after HEPE treatment [72]. Moreover, to further explore the apoptogenic capabilities of oxylipins [65], the activation of *caspase 3/7* and *caspase 8* genes was followed in sea urchin embryos treated with two PUAs (HD and OD) and four HEPEs (5-, 9-, 11- and 15-HEPE) [70]. In particular, both classes of compounds induced apoptosis, mostly at 9 and 24 hpf, detected by the luminometric assay and real time qPCR. Microscope observations showed that embryos subjected to PUA treatments were dead at 48 hpf, whereas HEPEs induced a developmental delay at both blastula and *pluteus* stages, confirming that PUAs greatly impacted sea urchin embryo development [70].

Several molecular approaches were also applied in order to explore the gene pathways activated by PUAs and HEPEs. Marrone et al. [66] revealed that the expression of sixteen genes, involved in stress response, skeletogenesis and development/differentiation processes, was significantly affected by DD, in a dose-dependent manner. In this study, the authors suggested that these genes are part of the “defensome”: genes and proteins integrated in a functional network able to protect an organism against natural toxins and xenobiotics [66]. The expression of these sixteen and other genes was also altered by HD and OD [67]. These targeted genes, examined by interactomic analysis (Ingenuity Pathway Analysis, IPA) were found functionally correlated to four *HUB* genes, *NF-κB*, *p53*, *δ-2-catenin* and *HIF1A*, which, in turn, were affected by PUA exposure [71]. In a similar study, IPA analysis was applied to further explore the molecular pathway involved in the response to PUAs in *P. lividus*. In particular, an additional twelve genes (*FOXA*, *FoxG*, *GFI-1*, *nodal*, *JNK*, *OneCut/Hnf6*, *TAK1*, *tcf4*, *TCF7*, *VEGF*, *Foxo* and *Jun*), linked to those isolated in previous studies [67,71], were also shown to modify their expression in sea urchin embryos treated with PUAs [73]. Molecular analyses using the primer pairs for the same genes analysed by Varrella et al. [67,71] and Ruocco et al. [73] revealed that 5- and 15-HEPEs had very few common molecular targets, with 5-HEPE switching on the highest number of genes, mainly at the early and swimming blastula stages [72].

Since marine organisms are normally exposed to LOX products as a whole, very recent studies were conducted to evaluate the potentially negative effect of PUAs and HEPEs mixtures on the sea urchin *P. lividus* [74,76,78]. Specifically, Ruocco et al. [76] showed that, by decreasing PUAs concentrations to one third of those used in individual tests (reported in Varrella et al. [67]), both binary and ternary mixtures were able to induce malformations in a synergic way, with the highest percentage of malformed plutei achieved in the case of 0.5 μM DD plus 1.0 μM HD at 48 hpf. A similar study [74] was done with combinations of the four HEPEs already tested separately in a previous study [72]. In particular, Albarano and co-workers observed several malformations that were much more severe compared to those reported in individual tests, revealing, also in this case, a synergic effect of these natural toxins. From the molecular viewpoint, these mixtures induced an additive effect when compared to experiments with single compounds [67,72], since a greater number of genes were affected [74,76]. Interestingly, PUA mixtures affected gene expression mainly at 48 hpf [76], while HEPEs were most effective in early developmental stages (particularly at 5 hpf) [74], confirming the inability of sea urchin embryos to recover after HEPE treatment [72].

The effects of oxylipin combinations on sea urchin embryos were further clarified by testing PUAs plus HEPEs mixtures [78]. Morphological observations revealed that these mixtures induced a stronger effect, compared to single compounds, with a dose-dependent developmental delay. Differently to individual tests, the high capability of PUAs to cause abnormalities was almost completely reverted by the presence of HEPEs in the same mixture. In fact, even if PUAs in individual tests resulted stronger than HEPEs, when in mixtures PUAs + HEPEs the effects of HEPEs diluted those of PUAs. In fact, in the first 48 hpf, oxylipin mixtures only induced developmental delay in sea urchin embryos and no malformed embryos were detected [78]. Moreover, IPA analysis led to the isolation of twelve new genes that were functionally correlated to eleven genes already identified in previous studies [67,71,73]. Real time qPCR analyses revealed that almost all of the genes, belonging to stress and developmental processes were significantly altered (>2-fold) [78]. Taken together, all these results strongly indicate that the delay observed in the early development of sea urchins exposed to oxylipin mixtures may be due to HEPEs, which act in an irreversible way, targeting many genes involved in skeletogenesis and development/differentiation processes already at the blastula stage.

All of the above studies reported the effects of in vitro tests on sea urchin eggs with commercially available pure molecules, but data from in vivo exposure to diatom-producing oxylipins are quite scarce. Gudimova et al. [68] conducted several tests incubating the eggs of the sea urchins *Strongylocentrotus droebachiensis* and *Echinus acutus* with the diatoms *Chaetoceros socialis*, *Skeletonema marinoi*, *Chaetocerus furcellatus*, *Attheya longicornis*, *Thalassiosira gravida* and *Porosira glacialis*. Specifically, to define the effects on sea urchin embryo development and survival, they used two diatom concentrations corresponding to the highest and lowest levels found during the spring bloom. At low (20 μg/L) and high (50 μg/L) concentrations, *S. marinoi* was the diatom causing the strongest impairment in the first cleavage of eggs of *S. droebachiensis* after 4 h of exposure and cell death in both sea urchins after 24 h of treatment [68]. The stronger impact of *S. marinoi* could be explained by its capability to release PUAs from cells before reaching the decline phase [79,80]. Feeding experiments with the same diatom species on *S. droebachiensis* plutei showed that the 4-arm plutei solely ingested *A. longicornis* species, the least harmful diatom in egg exposure experiments, while the other species triggered a high mortality rate (100% in the case of *T. gravida*). Regarding the six-arm plutei, no mortalities were recorded, revealing that probably the early stages of development were more sensitive to diatom toxins [68].

Very recently, feeding experiments were also conducted on adult *P. lividus* using four benthic diatom species in order to explore their negative impact on sea urchins compared to planktonic diatom species [75,77]. In particular, one-month of feeding on *Nanofrustulum shiloi*, *Cylindrotheca closterium* and *Diploneis* sp. induced malformations in sea urchin plutei spawned from diatom-fed individuals, with *N. shiloi* being the most toxic diet (55% of malformed plutei). The fourth species, *Cocconeis scutellum*, did not induce any effect on sea urchin offspring with a percentage of abnormal plutei very similar to controls (about 10%). De novo transcriptome approaches also revealed that benthic species affected several molecular pathways with very few common targets [75,77]. The highest activity detected in *N. shiloi* species may be explained by chemical analyses [22]. In fact, *N. shiloi* revealed a high content of both oxygenated fatty acids and PUAs, while *C. closterium* was less rich in oxylipins and produced mainly non-volatile oxygenated fatty acids. Interestingly, *Diploneis* sp., inducing about 40% of malformed plutei, exhibited several unknown compounds, probably related to a LOX-independent fatty acids metabolism [22]. According to feeding experiments, in which no negative effects were detected [77], a total absence of oxylipins was found in *C. scutellum* [22]. This result supports those by Zupo et al. [81] that post-larval feeding with *C. scutellum* induces only positive effects such as post-larvae settlement (about 63%) and survival.

Lipoxygenase activity has been reported in the sea urchin *Strongylocentrotus purpuratus*, leading to the formation of four hydroxyeicosanoids in homogenates of eggs, (11R)-hydroxy-5,8,12,14-ZZEZ-eicosatetraenoic acid and (12R)-hydroxy-5,8,10,14-ZZEZ eicosatetraenoic acid (from arachidonic acid) and the corresponding (11R)- and (12R)-hydroxy analogues of eicosapentaenoic acid [29,82,83]. No data are available on the sea urchin *P. lividus*. To date, gene sequencing confirms the presence of lipoxygenases in the sea urchin *S. purpuratus* and in other marine invertebrates but their mechanism of action is still unknown. Furthermore, the presence of lipoxygenases in the genome of the sea urchin does not imply that these genes are actually expressed and that they have the same function as in diatoms. In contrast, given the wide variety of ecological roles of lipoxygenases in various organisms (from terrestrial plants to marine animals and microalgae), it is likely that lipoxygenase activity reported for some sea urchins has a physiologic role that is quite different from that of marine diatoms.

### 2.2. Marine Copepods

As opposed to the literature on sea urchin–oxylipin interactions, few studies have reported on the effects of exposure to pure molecules in copepods. The effects of diatom-derived oxylipins were mostly evaluated by feeding adults and/or larvae with specific diatom species (mostly the PUA-producing *S. marinoi*) for which a LOX activity was already described (Table 2).

Diets of two bloom-forming algae, the diatom *S. marinoi* and the dinoflagellate *Scrippsiella hangoei*, were evaluated on egg production in the copepod *Acartia bifilosa*. Copepods produced the highest number of eggs with the *S. hangoei* diet, whereas *S. marinoi* was the most effective in impairing copepod reproduction [91]. The effect of three *S. marinoi* strains producing different quantities of PUAs were assessed in three common planktonic copepods *Acartia tonsa, Pseudocalanus elongatus* and *Temora longicornis* [84]. The hatching success of *A. tonsa* was almost the same for all diets until Day 6, after which a significant decrease (less than 30% of hatched nauplii) was observed with strain GF04-9B. A reduction in the number of hatched nauplii was also observed in *P. elongatus* and *T. longicornis* fed with the GF04-9B strain. Since the most toxic strain was not the richest in terms of PUAs production, no significant correlation was found between the impairment of embryo development and the abundance of PUAs [84].

Monoalgal and mixed diets of *Prorocentrum minimum* (control diet), *S. marinoi* (positive control) and *T. rotula* were also evaluated on the development and sex differentiation of the copepod *Temora stylifera* [86]. Mortality rates were higher in *Temora stylifera* fed with *T. rotula* compared to *P. minimum* plus *T. rotula*, suggesting that a beneficial food was able to dilute the negative effect of a toxic diatom. On the contrary, no significant differences were recorded between *S. marinoi* and mixed diets (*S. marinoi* plus *P. minimum*), in both maternal and larval diets. In particular, offspring generated by females fed with *P. minimum*/*S. marinoi*, and successively raised on *P. minimum*/*S. marinoi* or mixed diets (*P. minimum* plus *S. marinoi*), arrested their development within a few days. Furthermore, larval feeding strongly affected the final sex ratio of *T. stylifera* in both *S. marinoi* and *T. rotula* diets [86]. Maternal and larval diets were also tested in the copepod *Paracartia latisetosa* analysing the effect of *S. marinoi* ingestion compared to the control *P. minimum* [96]. Feeding of both adults and offspring on *S. marinoi* induced the lowest egg production and viability, as well as a strong delay in embryo development. Moreover, mixed diets revealed that nauplii were more sensitive to the PUA-producing diatom *S. marinoi*. In fact, development to adulthood (up to eleven days) was observed when nauplii were reared on *P. minimum* and spawned from females fed on *S. marinoi*, whereas, in the opposite condition, a blockage of naupliar development was observed [96].

Three PUAs-producing diatom species were tested on the copepod *T. stylifera* by measuring egg production and hatching success [85]. In particular, these authors analysed the effects of *S. marinoi*, *Thalassiosira rotula* Strain CCMP 1647 (TR1), *T. rotula* Strain CCMP 1018 (TR2) and a species that did not exhibit PUAs activity, *Skeletonema pseudocostatum*. All diatoms reduced egg production rates and hatching success compared to diets of the dinoflagellate *P. minimum* (control diet). Surprisingly, *S. pseudocostatum*, the non-PUA producing species, together with *S. marinoi* induced the strongest toxicity. Moreover, 88% of nauplii that hatched from copepods fed with *S. pseudocostatum*, were TUNEL-positive after 48 h, revealing that some apoptotic events had occurred. The effects of this diatom were attributed to other oxylipins belonging to oxygenated fatty acids (15*S*-HEPE, 13,14-HEpETE and 15-oxoacid) reported in the same study [85]. *T. stylifera* was also fed with two diatoms, the PUAs-producing *S. marinoi* and *P. delicatissima* [87], in which an oxygenated fatty acid (15*S*-HEPE) was previously described [32]. Both diets affected egg production rates (less than 10%) and viability, which declined more dramatically with a diet of *S. marinoi*. Female survival was slightly reduced after 15 days of feeding (93.7% with SKE and 94.7% with *P. delicatissima*), reaching more significant values at the end of the experiment (75% with *S. marinoi*). Several TUNEL-positive regions were also observed in nauplii hatched after *T. styifera* feeding with both diatoms, revealing that, probably, 15*S*-HEPE could also trigger some apoptotic events and negatively affect the reproductive capability of copepods [87]. Comparative studies of diatom blooms in 2004 and 2005 corroborated this hypothesis [40]. In particular, the hatching rate in both *Acartia clausi* and *Calanus helgolandicus* decreased from 2004 to 2005 (~80% in 2004 and ~60% in 2005), when the abundance of oxygenated fatty acids was significantly higher [40].

More recently, diets with two diatom-producing oxygenated fatty acids, *Chaetoceros muelleri* and *Nitzschia closterium f. minutissima* [22,90,98], were tested on the planktonic copepods *Acartia pacifica* and *Pseudodiaptomus annandalei* and the benthic species *Tigriopus japonicus* [97]. These studies showed that the effect of diatoms was species-specific, since a diet with *N. closterium f. minutissima* was found to be particularly unsuitable only for the copepod *A. pacifica*. In fact, at all concentrations tested, this copepod was not able to complete naupliar development, whereas *P. annandalei* nauplii normally developed to adults. Furthermore, all diatom species analysed did not have a significant impact on the development of the benthic copepod *Tigriopus japonicus* [97]. A species-specific interaction could explain the contrasting results of some mesocosm experiments, showing no effects of the PUA-producing diatom *S. marinoi* on the reproduction of the copepod *Calanus finmarchicus* [88]. In particular, diets with low and high concentrations of *S. marinoi* supplied with nitrates, phosphates and silicates did not affect hatching success and the survival of nauplii [88].

The first molecular studies on copepods investigated the effects of diets of the diatom *S. marinoi* on the copepod *C. helgolandicus* compared to control diets with the dinoflagellate *P. minimum* and the green alga *Rhodomonas baltica*, which does not produce oxylipins [89]. The expression levels of two mitochondrial subunits were found significantly altered with *S. marinoi* diets, with the downregulation of α- and β-tubulin. Conversely, *P. minimum* and *R. baltica* diets induced no significant changes in the expression of α- and β-tubulin [89]. A similar study showed that a monoalgal diet of *S. marinoi* was sufficient to downregulate a pool of genes involved in stress response, defence system and detoxification in the copepod *C. helgolandicus* [90]. These data were compared to a diet of *C. socialis* that produces low amounts of oxylipins that did not affect the expression levels of genes under analysis. The same authors performed a comparative study between two populations of *C. helgolandicus* from the Swedish Western Coast and the Mediterranean Sea [92]. *S. marinoi* diets altered the expression of detoxification enzymes and proteins involved in apoptosis and cell cycle progression, with the Mediterranean population being more susceptible after 24 and 48 h of feeding [92]. The molecular effects of *S. marinoi* diets on a different *Calanus* species (*C. sinicus*) were also analysed, using *R. baltica* diet as a control [95]. Although a significant downregulation of genes involved in defence and detoxification systems was detected after five days of feeding, this copepod species was more resistant than the congeneric species *C. helgolandicus*. Field studies evaluated the impact of spring blooms in Goro and Rimini stations (Adriatic Sea) on the reproductive success of the copepod *C. helgolandicus* [41]. Interestingly, they showed that the area with the lowest egg production and hatching success corresponded with high oxylipin abundance. Furthermore, copepods collected in both sites had a significant upregulation of stress-related genes, such as heat shock proteins, catalase, S-transferase glutathione and aldehyde dehydrogenase, compared to laboratory conditions in which copepods were fed with the dinoflagellate *P. minimum* [41]. Molecular studies were also performed by Carotenuto et al. [93], who generated two Expressed Sequence Tags (ESTs) libraries of the copepod *C. helgolandicus* fed on both *S. marinoi* and the control *R. baltica*, using suppression subtractive hybridisation (SSH). Comparison of SSH libraries revealed that some biological processes, such as response to stimuli, signal transduction and protein folding were over-expressed in copepods fed with *S. marinoi*. These results were also validated by real time qPCR [93].

Feeding investigations were also combined to in vitro exposure of ripe females with DD, HD and 15*S*-HEPE, at a concentration range of 1.0–20 μg/mL [87]. All compounds induced a similar dose-dependent reduction in hatching success in *T. stylifera* nauplii, with the two PUAs inducing stronger effects compared to 15*S*-HEPE. This result was also confirmed using the TUNEL assay, showing that apoptotic tissues were visible in treatments with 15*S*-HEPE at a concentration ten times greater than PUAs [87]. A similar study tested various concentrations of DD (0.5–12 μg/mL) on adults of the copepod *T. stylifera* [24]. In particular, although egg production rates indicated a dose-dependent increase, hatching time and success were significantly altered with only 54% of hatched nauplii at 2 μg/mL. Among nauplii, the majority displayed apoptotic features, detected using the TUNEL-assay. In addition, DD at a concentration greater than 3 μg/mL induced a higher mortality in males and females compared to the controls. Moreover, odour choice experiments revealed that some unknown mechanisms stimulate copepods towards DD, acting as chemical signals [24]. Dhanker et al. [94] also studied the effects of exposure to several concentrations of DD (0.75, 1.5, 3 and 4.5 mM) on the copepod *Pseudodiaptomus annandalei*. As a result, DD significantly reduced female survival and naupliar production in a dose-dependent manner. Furthermore, this PUA induced high mortalities in nauplii and significantly delayed development times [94].

### 2.3. Miscellaneous

A few studies have explored the possible negative effects of oxylipins on other organisms in the last ten years (Table 3).

In 2011, the effects of different DD concentrations were evaluated on the reproductive success and life cycle of the polychaete *Nereis virens* [99]. In particular, the authors showed that DD treatments were able to cause a strong decrease in fertilisation rate and larval viability, together with a significant impairment of sperm motility in a dose- and time-dependent manner.

Moreover, Comet assays revealed visible DNA damage in treated sperm, with results even higher when compared to those induced with copper sulphate [99].

The tunicate *Ciona intestinalis* was used to test the effects of increasing DD concentrations on post-hatched embryos at three different times after hatching (early, middle and late larval stages) [100]. At lower concentrations (0.8 μM), the authors observed a significant delay in settlement time and metamorphosis after 24 h of treatment when DD was added to middle and late larval stages. At higher concentrations (8.9 μM), metamorphosis was completely blocked. Moreover, the 2,3-diaminonaphthalene (DAN) assay showed a decrease in endogenous NO that was confirmed by molecular experiments, revealing that middle larvae (20–21 hpf) treated with DD displayed no significant variation in the expression of the *NO synthase* (*NOS*) gene. NO levels were also shown to be finely regulated by several genes involved in redox homeostasis, such as the *glutamate–cysteine ligase regulatory subunit* (*gclm*) and *gamma-glutamyl transpeptidase* (*ggt*) genes, which were found significantly upregulated in treated larvae [100]. Since the ERK pathway is known to promote metamorphosis, real time qPCR was also applied to evaluate the expression of genes implicated in the ERK pathway. In particular, relative expression analysis indicated that DD was able to inhibit metamorphosis by inducing a strong upregulation of a specific *map kinase phosphatases* (*mkp1*), whose activity is able to block ERK signalling [100]. In a similar study, *C. intestinalis* oocytes were treated with increasing concentrations of DD to follow embryo development [102]. Morphological analyses indicated a dose-dependent developmental delay and aberrations mainly affecting the larval tail. Furthermore, a reduction in hatching capabilities was recorded, with a very low percentage of hatched larvae (less than 20%) at the highest concentration tested. From the molecular point of view, the authors showed that DD exposure was able to target many genes involved in developmental and stress response processes [101]. DD effects were also evaluated on the tunicate *Oikopleura dioica* [103]. In particular, the authors showed that *O. dioica* embryos, deriving from eggs treated with different concentrations of DD (0.25–2.0 μg/mL), evolved dose-dependent aberrations*,* affecting morphogenesis, midline convergence and tail elongation processes. At higher DD concentrations (>2.5 μg/mL), embryo abnormalities were more severe, with a complete blockage of first cleavage. Moreover, the authors validated these data by whole-mount in situ hybridisation, showing that DD treatments were able to cause a systematic delay in the expression of many developmental genes [103]. Finally, tests with crude extracts of oxylipin-producing diatoms, *S. marinoi* and *Chaetoceros affinis*, on the eggs of *O. dioica* showed that natural PUAs and/or oxygenated fatty acids were able to induce the same aberrations as those observed with DD treatments [103].

Since diatoms constitute a great source of nutrients for small organisms living in the zooplankton, Lavrentyev et al. [102] tested HD and OD mixtures at different concentrations on several microzooplankton species, to define the impact of these natural toxicants on their development. Specifically, PUAs treatments induced variable developmental delays in a dose- and species-dependent manner. In fact, the results showed a negative effect on some ciliates and dinoflagellates, whereas other species were not affected or their response was activated only at the highest concentrations [102].

Very recently, the impact of a simulated marine warming environment in combination to DD exposure was evaluated on larval fitness of the fish cobia *Rachycentron canadum* [104]. Survival slightly decreased (16%) after exposure to high temperature (29 °C) and 0.5 μM DD, reaching higher values when these disturbances were combined. In fact, when PUA-treated larvae were exposed to high temperatures, the percentage of viable larvae was reduced to about 60%, revealing a synergistic effect [104].

## 3. Oxylipins as Cell Signalling Molecules in Diatom Communities

As mentioned above, oxylipins may act as toxic compounds regulating population dynamics at the end of blooms when stress conditions increase and nutrient availability is quite limited [59,62,105]. It has been hypothesised that an accurate mechanism of bloom regulation could be activated [59]. In fact, 2*E*,4*E*-decadienal was found to trigger programmed cell death (PCD) in diatom cells by inducing the release of intracellular calcium and a consequent increase in NO levels. Surprisingly, when treatments were applied at sub-lethal concentrations (660 nM), diatoms became resistant to higher successive doses (13.2 μM), without the activation of PCD and a total unresponsive calcium cascade [59]. In addition to oxylipins, the end of bloom events was also associated to bacterial or viral infections controlling phytoplankton dynamics [106,107]. Recently, a chemical defence role against some bacterial species that leads to cell lysis in microalgal blooms was proposed. In particular, when a diatom-producing HEPEs, *Chaetoceros didymus*, was co-cultured with an algicidal bacteria, *Kordia algicida*, a significant decrease in bacterial growth and cell lysis was detected. Chemical analyses of culture media confirmed a huge production of HEPEs, particularly 15-HEPE, which might be considered the main HEPE responsible for *C. didymus* protection during bloom events [108]. Interestingly, a surprising plasticity of PUAs production was found. Laboratory cultures with low silicate concentrations revealed high PUA levels in the medium, which corresponded to a poor degree of cell wall silicification, thus suggesting that a probable switch between chemical and mechanical defence was finely regulated [109].

The first evidence indicating that PUAs were produced not only after wound-activation but also to regulate bloom events and cell-to-cell communication was published by Casotti et al. [53]. Moreover, Vidoudez and Pohnert [79] observed that HD and OD concentrations increased in culture media of *S. marinoi* by Day 21. Moreover, the addition of these PUAs during different stages of growth revealed a strong decrease in cell numbers when added at the stationary and declining phases, possibly indicating that PUAs act as intra-population signals able to regulate bloom events.

Many studies have also corroborated the hypothesis of a possible role of oxylipins as allelochemicals. For instance, a negative effect of DD was observed in cell growth and viability of the diatom *Thalassiosira weissflogii*; incubation with decadienal decreased the growth rate in a dose- and time-dependent manner, with dead cells displaying the typical characteristics of apoptotic events, including cell shrinkage and DNA damage [53]. Allelopathy was also shown when the three PUAs, DD, HD and OD were tested on the prymnesiophyte *Isochrysis galbana*, the chlorophyte *Tetraselmis suecica* and the diatom *S. marinoi* [54]. Flow cytometry experiments revealed that PUAs altered the morphology of all species, with *S. marinoi* being the most resistant to oxylipins toxicity [54]. The same PUAs, in single and mixture experiments, were tested on *S. marinoi* and *Phaeodactylum tricornutum* diatom cultures [57]. In particular, the diatom *S. marinoi*, exerted a reduction in NO levels with a parallel ROS increase when treated with HD and OD, two compounds produced by this diatom species. Since NO levels increased significantly in *P. tricornutum* exposed to DD, a species-specific response was proposed, in which *S. marinoi* perceived HD and OD as intra-population signals, while *P. tricornutum* recognised them as allelochemicals [57]. The same authors further demonstrated that, in addition to free radical species, PUAs response involved the generation of O_2_^−^ and superoxide dismutase (SOD) activity, which was confirmed by measuring the accumulation H_2_O_2_ [110]. The allelopathic potential of diatom oxylipins was also investigated in the invasive dinoflagellate *Ostreopsis* cf. *ovata* [111,112]. The exposure to DD, HD and OD induced a growth inhibition and cell abnormalities, with higher effects triggered by the long-chained aldehyde DD, comparing to the shorter PUAs [111].

Some mechanisms of auto-allelopathy have also been recorded under low-nutrient conditions, which normally occur at the end of blooms [113]. Recently, auto-allelopathic interactions were observed in treatments with the hydroxyacid 15-HEPE purified from the medium of a *S. costatum* strain [114]. In particular, when 15-HEPE was administered to a culture of the dinoflagellate *Alexandrium minutum*, no inhibitory effect was detected, while a strong decrease in growth rate was measured in *S. costatum* cultures.

Diatom-derived oxylipins were also involved in the regulation of bacteria–phytoplankton community dynamics [51], influencing cell growth and species composition that, in some cases, were hypothesised to be implicated in combination with additional molecules involved in diatom–bacteria interactions [115]. The PUAs DD, HD and OD were tested on 33 marine bacterial strains, including several species isolated from a bloom of the PUA-producing diatom *S. marinoi* [55]. Since a visible resistance of bloom species was detected, PUAs were confirmed to be fundamental in shaping associated bacterial communities, particularly at the end of bloom events when senescence and declining nutrient concentrations favour an increase in the production of PUAs [55]. The same PUAs were confirmed to promote the growth of PUAs-resistant species when tested on a natural bacterial community. This result is of significant ecological relevance, since resistance to PUAs toxicity could provide a precious advantage to bacterial communities, by increasing the possibility of using the organic matter released by diatoms [56]. The mechanism of action of PUAs entry into bacterial cells was later described as the strong accumulation on cytoplasmic membranes due to their hydrophobic properties [116].

PUAs also play a critical role in sinking processes and particulate organic carbon (POC) exportation from swallow to deeper waters [58]. In particular, incubation of PUAs at low concentrations (1–10 μM) was found to induce the remineralisation of organic matter and the growth of POC associated bacteria (about 50% greater than control), together with a significant change in bacterial community structure. On the contrary, at higher concentrations (100 μM), bacterial cell abundance and metabolism was significantly lower. These results led to the conclusion that, on inter-annual timescales, PUAs decrease the efficiency of POC export from surface to deeper waters and, consequently, induce the retention in shallow waters of phosphorus and other nutrients, which are, in turn, available to primary producers [58]. The influence of PUAs on carbon export in marine environments was further explored in a mesocosm experiment [117]. Transparent exopolymeric particles that spontaneously form through abiotic processes were found to be critical for particle aggregation and organic carbon flux from shallow to deeper zones. The addition of a mix of three PUAs (DD, HD and OD) during the exponential phase of an artificial bloom of *T. rotula*, significantly increased the quantity of dissolved organic carbon (DOC) and the abundance of exopolymeric particles with respect to the control. Since exopolymeric particles levels and size significantly increased at the end of the bloom, PUAs were confirmed to enhance the export of organic carbon, altering food web structure and the consequent size and distribution of available food particles [117]. Contrary to the results of Edwards et al. [58], the abundance of free bacteria was almost the same at the end of the experiment, suggesting that PUAs did not influence the bacterial community [117]. Recently, this latter observation was confirmed, since treatments with heptadienal and octadienal on two strains of *S. marinoi*, a PUA- and a non-PUA producer, showed no significant differences in bacterial communities between the two cultures [118]. Overall, these contrasting results reinforce the idea, previously suggested by Paul et al. [115], that PUAs could act in a more complex manner, where additional chemical mediators are also involved.

## 4. Biotechnological Applications of Oxylipins

Thus far, few studies have focused on the possible biotechnological applications of oxylipins, including anti-cancer, anti-bacterial, anti-fungal and anti-parasitic activities (Table 4). The first study to suggest the possible anti-cancer activity of oxylipins was published by Miralto et al. [7]. In particular, MTT (thiazolyl blue) and TUNEL assays revealed antiproliferative and apoptotic activities of the diatom-derived PUAs, DD and 2*E*,4E/*Z*,7*Z*-decatrienal (DT), in human colon adenocarcinoma cell lines Caco2 [7].

Afterwards, other studies have tried to explore these interesting findings. The extracts and fractions from *Cocconeis scutellum parva*, an oxylipin-producing diatom [125], were tested on several cancer cell lines [121]. In particular, an EPA-enriched fraction from the diethyl-ether extract was the most active against breast carcinoma (BT20) cells, triggering up to 89.2% apoptosis. Furthermore, a dose-dependent decrease of BT20 cell viability was associated to the activation of caspases-8 and caspase-3 and the blockage of cell cycle progression from S to G2-M phases [121]. Several concentrations of three synthetic PUAs, DD, HD and OD, were also tested on two adenocarcinoma cell lines (A549 and COLO 205) [63]. MTT assays indicated that DD had the highest anti-proliferative activity on these two cancer cell lines but was not active on the normal lung/brunch epithelial BEAS-2B cell line. Moreover, immunoblotting analyses showed that all PUAs were able to activate the apoptotic extrinsic pathway mediated by Tumour Necrosis Factor Receptor 1 (TNFR1) and Fas Associated Death Domain (FADD). These results were confirmed by molecular approaches. In fact, DD and HD induced a significant upregulation of a pool of genes involved in apoptosis such as, *TNFRSF1A* and *TNFRSF1B* (coding for the two receptors TNFR1 and TNFR2), *FADD*, *caspase-3* and *AIFM1*. On the contrary, OD showed a lower activity, since no variation in gene expression was recorded. Finally, the apoptotic events were also evaluated by flow cytometry techniques, revealing, once again, a lower anticancer activity in OD treatments [63]. An ambiguous result was achieved when 32 microalgae species were screened for anti-inflammatory, antitumor, antibacterial, antidiabetic and antioxidant activities [123]. In fact, of two clones of the diatom *S. marinoi* tested, only one had anti-cancer activity against human melanoma cells (A2058), depending on nutrient conditions. Moreover, since the antibacterial activity on *Staphylococcus aureus* was found in both clones but only in nitrogen-starvation conditions, the authors suggested that probably oxylipins are not responsible for such activity [123]. The same authors found an anti-tuberculosis activity from the extracts of the PUA-producing diatom *S. costatum* together with *Chaetoceros pseudocurvisetus*. In particular, these algae were found to be active against *Mycobacterium tuberculosis* and *M. bovis* only in phosphate-starvation culturing condition [124].

Possible nutraceutical applications of diatom-derived oxylipins were also proposed [122]. In particular, the effects of an oxylipin-containing lyophilised (OLM) biomass from the freshwater alga *Chlamydomonas debaryana* were evaluated on a recurrent 2,4,6-trinitrobenzenesulfonic acid (TNBS)-induced colitis mice model. The oral administration of OLM lyophilised induced anti-inflammatory activities with a significant decrease of pro-inflammatory cytokines (TNF-α, IL-1β, IL-6 and IL-17), iNOS, COX-2 and NF-κB, together with the increase of PPAR-γ levels [122].

In some cases, oxylipins were also used as possible anti-parasitic agents useful to treat diseases that commonly occur in aquaculture practices. For instance, Simon et al. [119] tested the effects of different decadienal concentrations on the survival and growth of two polychaetae larvae, *Boccardia proboscidea* and *Terebrasabella heterouncinata,* that normally infest breeding of the abalone *Haliotis midae.* Specifically, they observed a dose- and time-dependent negative impact on larval development and survival of both polychaetes with a higher sensitivity of *T. heterouncinata* [119]. In a different study, infected salmon (*Salmo salar*) with the parasite *Caligus rogercresseyi* were treated with DD, using this aldehyde as a food supply [120]. In particular, no significant toxicity was observed in histopathological sections of salmon injected with increasing concentrations of decadienal in brain, intestine, skin, liver and muscle tissue. Moreover, DD feeding at non-toxic concentrations was able to impair the reproductive capability of *C. rogercresseyi* by decreasing the number of mature females and eggs [120].

Another key issue is the influence of symbiotic bacteria on the ecology, physiology and the biotechnological potential of diatoms. Diatoms and bacteria have co-occurred in common habitats for more than 200 million years [126]. As a result, hundreds of genes found in certain diatoms species, have been acquired from bacteria [127,128]. During evolution, diatoms have established a species-specific relationship with bacteria due to a strong cooperation that favours one another. In fact, the oxygen coming from photosynthesis is used for bacterial degradation of organic matter, while bacteria release CO_2_ through remineralisation processes to facilitate the complete photosynthetic cycle [129]. Interestingly, a functional carbon flux between diatoms and bacteria has been observed since some bacterial species have been found as intermediate providers for the biosynthesis of bioactive metabolites [130]. Overall, co-occurring bacteria promote the growth of diatom cells, influencing their metabolism and improving their biotechnological potential [131,132,133,134,135,136].

## 5. Concluding Remarks

The ecological role of diatom-derived oxylipins is extremely complex, since it consists of multiple functions affecting population dynamics of aquatic environments.

As reported above, some oxylipins are toxic compounds produced by diatoms that negatively affect the reproductive success of several marine invertebrate consumers. In fact, oxylipins, particularly PUAs, act as chemical deterrents against grazers, interfering with the reproductive success of some marine invertebrates, starting from gamete viability, fertilisation processes, embryogenesis until larval fitness. Most studies in the last ten years mainly investigated their effects on sea urchins and copepods. Given the importance of diatom blooms in marine environments and the ecological implications, it would be interesting to extend these studies to other marine invertebrates, in order to better understand the mechanisms of response to oxylipins.

**Figure 3:**
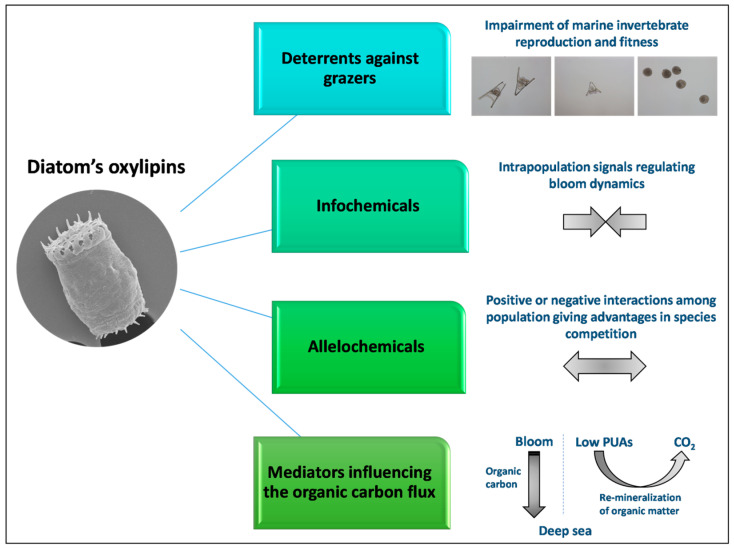
Oxylipins in aquatic environments can act as deterrents against grazers, info-chemicals, allelochemicals and mediators that influence carbon recycling.

Moreover, in addition to their synthesis upon wound-activation, these oxygenated fatty acid derivatives can also be actively produced by intact diatoms cells through mechanisms that are still unknown. The role of these small chemical mediators is to regulate cell–cell communication within the same species and among different species, influencing the structure and the composition of phytoplankton communities (Figure 3).

Few studies have evaluated the possible biotechnological applications of oxylipins; this is probably due to a low chemical stability that makes them quite difficult to manipulate in laboratory conditions. Nevertheless, interesting bioactivities have been reported, ranging from anticancer to antibacterial capabilities. Overall, the ecological role of diatom oxylipins and their potential pharmacological applications deserve further investigations.

## Figures and Tables

**Table 1:** Species, oxylipins or diatom diets, concentrations tested and morphological and molecular effects (highlighting the most representative results) reported in the literature on sea urchins during 2010–2020. Abbreviations: DD, 2*E*,4*E*-decadienal; HD, 2*E*,4*E*-heptadienal; OD, 2*E*,4*E*-octadienal; OT, 2*E*,4*E*,7*Z*-octatrienal; 5-HEPE, 5-hydroxy-6*E*,8*Z*,11*Z*,14*Z*,17*Z*-eicosapentaenoic acid; 9-HEPE, 9-hydroxy-5*Z*,7*E*,11*Z*,14*Z*,17*Z*-eicosapentaenoic acid; 11-HEPE, 11-hydroxy-5*Z*,8*Z*,12*E*,14*Z*,17*Z*-eicosapentaenoic acid; 15-HEPE, 15-hydroxy-5*Z*,8*Z*,11*Z*,13*E*,17*Z*-eicosapentaenoic acid.

Species	Oxylipins (μM)/Diatom	Morphological Effects	Molecular Effects	Reference
*P. lividus*	DD, HD, OD and OT (0.658–32)	Cleavage inhibition; Malformed plutei with decadienal	Not detected	[64]
*P. lividus*	DD (0.002–0.03)	Increase of endogenous NO levels and consequently apoptosis induction	Upregulation of *hsp70* and *caspase-8*; downregulation of *NOS*	[65]
*P. lividus*	DD (0.001–0.0023)	Differentially expressed genes with dose dependent effect	Upregulation of *hsp70*, *hsp56*, *hsp60*, *hat, BP10, 14-3-3ε, p38 MAPK, GS* and *MTase*; downregulation of *sox9*, *uni*, *SM30, Nec* and *SM50*	[66]
*P. lividus*	DD (0.5–2.5), HD (1.0–6.0) and OD (2.0–9.0)	Dose-dependent malformations of sea urchin plutei	Upregulation of *hsp70*, *hat*; downregulation of *SM50*, *Wnt6*, *MT4* and *MT6*	[67]
*S. droebachiensis E. acutus*	*C. socialis*, *S. marinoi*, *C. furcellatus*, *A. longicornis*, *T. gravida* and *P. glacialis* (20 and 50 μg/L)	Blocks of first mitotic division after 4 hpf with *S. marinoi*	Not detected	[68]
*E. mathaei*	DD (0.4–1.2), HD (0.5–1.5) and OD (0.6–1.7)	Dose-dependent malformations of sea urchin plutei	Not detected	[69]
*P. lividus*	5-, 9-, 11-, 15-HEPE (100), DD (3.3), HD (9.0) and OD (11.0)	Impairment at blastula and pluteus stage with PUAs and HEPEs	Activation of *caspase-8* and *caspase-3/7*	[70]
*P. lividus*	DD (1.0–2.3), HD (2.0–6.0) and OD (2.5–8.0)	Not detected	Upregulation of *MTase* and *p38 MAPK*; downregulation of *MT6*, *CAT Alix* and *SM50*	[71]
*P. lividus*	5- and 15-HEPE (6–30)	Dose-dependent malformations of sea urchin plutei	Upregulation of *hsp70*, *hsp56*, *14-3-3ε*, *Blimp* and *MT5*; downregulation of *HIF1A* and *SM50*	[72]
*P. lividus*	DD (1.6), HD (3.0) and OD (4.5)	Not detected	Upregulation of *Jun* and *Foxo*	[73]
*P. lividus*	Mixture of 5-, 9-, 11-, 15-HEPEs (1.0–7.0)	Synergic effect of HEPEs	Downregulation of *MTase*, *p38 MAPK* and *Alix*	[74]
*P. lividus*	*N. shiloi* (1.8) and *C. closterium* (1.6)	Malformed plutei	Upregulation of *Blimp*, *hsp70*, hsp60, *GS*, *cytb*, *14-3-3ε*, *Nec*, *p19*, *jun, Blimp*, *Wnt6*, *nodal*, *FoxG*, *Foxo*, *OneCut*, *MT*, *CAT*, *MDR1*; downregulation of *MTase*, *p53*, *HIF1A*, *SM30*, *BMP5/7*, *uni*, *FOXA*, *GFI1*, *δ-2-catenin*, *VEGF* and *MT8*	[75]
*P. lividus*	Mixture of DD (0.5), HD (1.0) and OD (1.5)	Synergic effect of PUAs	Downregulation of *cytb, caspase-8*, *Alix*, *δ-2-catenin*, *tcf4, GFI1*, *OneCut*, *TAK1* and *MT7*	[76]
*P. lividus*	*C. scutellum* (1.5) and *Diploneis* sp. (1.6)	Malformed plutei with *Diploneis* sp.	Upregulation of *p53*, *GS*, *Alix*, *Wnt5*, *NF-kB*, *ERCC3*, *p16*, *MT*, *CAT* and *MDR1;* downregulation of *δ-2-catenin*, *hsp70*, *hsp60*, *tcf4* and *MT8*	[77]
*P. lividus*	Mixture of PUAs (DD 0.3, HD 0.7 and OD 1) and HEPEs (1.6)	Higher morphological effects than those detected with individual oxylipins and PUAs/HEPEs mixtures	Upregulation of *ADMP2, Delta, Goosecoid, KIF19, jun* and *CAT*; downregulation of *ARF1*, *GS*, *HIF1A* and *sox9*	[78]

**Table 2:** Species, oxylipins or diatom diets, concentrations tested and morphological and molecular effects reported in the last ten years on copepods. Abbreviations: DD, 2*E*,4*E*-decadienal; HD, 2*E*,4*E*-heptadienal; 15-HEPE, 15-hydroxy-5*Z*,8*Z*,11*Z*,13*E*,17*Z*-eicosapentaenoic acid; PUAs, polyunsaturated aldehydes.

Species	Oxylipins/Diatom	Morphological Effects	Molecular Effects	Reference
*A. tonsa* *P. elongates* *T. longicornis*	*S. marinoi* GF04-9B (264 μm^3^), GF04-1G (622 μm^3^) and GF04-7J (141 μm^3^)	Reduction of egg production and hatching success	Not detected	[84]
*T. stylifera*	*S. marinoi* and *S. pseudocostatum* (60*10^3^ cells/mL), *T. rotula* CCMP1647 and CCMP1018 (8*10^3^ cells/mL)	Reduction of egg production and viability, naupliar and female survival	Not detected	[85]
*T. stylifera*	*T. rotula* (2036 μm^3^) and *S. marinoi* (196 μm^3^)	Increase of mortality	Not detected	[86]
*T. stylifera*	15-HEPE, DD and HD (1.0 to 20 μg/mL)	Reduction of egg production, naupliar and female survival	Not detected	[87]
*C. finmarchicus*	*S. marinoi* G4 (400 cells/mL and 1000 cells/mL)	No effect on hatching success and naupliar survival	Not detected	[88]
*C. helgolandicus*	*S. marinoi* (45,000–60,000 cells/mL)	Not detected	Downregulation of *ATUB* and *BTUB*	[89]
*C. helgolandicus*	*S. marinoi* (45,000–60,000 cells/mL) and *C. socialis* (48,000–58,000 cells/mL)	Not detected	Upregulation of *CYP4*; downregulation of *GST*, *GSH-S*, *SOD*, *ALDH6*, *ALDH2*, *CARP*, *CAS*, *IAP, ATUB*	[90]
*A. bifilosa*	*S. marinoi* (41.6 μgC/cell)	Reduction of egg production	Not detected	[91]
*C. helgolandicus*	*S. marinoi* (45,000–6,0000 cells/mL)	Not detected	Upregulation of *HSP70*, *GST*, *SOD*, *ALDH3*, *ALDH8*, *ALDH9*, *BTUB*	[92]
*T. stylifera*	DD (0.5 to 2 μg/mL)	Reduction of egg production, hatching success and increase of mortality	Not detected	[24]
*C. helgolandicus*	*S. marinoi* (45,000 cells/mL)	Not detected	Upregulation of *HSP70*, *cyclin B1*, *glicoprotein 93*, *chaperonin-subunit ETA*, *diphosphate kinase*, *finger protein 121*,*14-3-3-protein*, *superoxide dismutase*; downregulation of *proteasome subunit*	[93]
*P. annandalei*	DD (0.75 to 4.5 μM)	Dose-dependent reduction of female survival and nauplii production	Not detected	[94]
*A. clausi* *C. helgolandicus*	PUAs (0.97 μg/mg protein in 2004 and 1.2 μg/mg protein in 2005) and oxygenated fatty acids (3.6 μg/mg protein in 2004 and 14 μg/mg protein in 2005)	Reduction of egg production and hatching success	Not detected	[40]
*C. sinicus*	*S. marinoi* (45,000 cells/mL)	Not detected	Upregulation of *ALDH2*, *ALDH8*, *ALDH9*, *SOD*, *GSH-S*, *GST*, *CAS*, *CARP*; downregulation of *HSP70*	[95]
*P. latisetosa*	*S. marinoi* (10^4^ to 10^6^ cells/mL)	Reduction of egg production, hatching success and incomplete naupliar development	Not detected	[96]
*C. helgolandicus*	oxygenated fatty acids (0.001 to 1389.13 ng/mg)	Reduction of egg production and hatching success	Upregulation of *ALDH8*, *ALDH7*, *ALDH6*, *CAT*, *GST*, *HSP40*, *HSP70*; downregulation of *CAS*, *CARP*, *BTUB*, *ATUB*, *ALDH3*, *SOD*, *CYP*	[41]
*T. japonicus* *A. pacifica* *P. annandalei*	*C. muelleri* and *N. closterium f. minutissima* (0.35 to 17.00 μgC/mL for *T. japonicus* and 0.35 to 8.5 μgC/mL for *A. pacifica* and *P. annandalei*)	Incomplete naupliar development	Not detected	[97]

**Table 3:** Species, oxylipins or diatom diets, concentrations tested and morphological and molecular effects reported in other marine invertebrates from 2010 to 2020. Abbreviations: DD, 2*E*,4*E*-decadienal; HD, 2*E*,4*E*-heptadienal; OD, 2*E*,4*E*-octadienal.

Species	Oxylipins (μM)/Extract	Morphological Effects	Molecular Effects	Reference
*N. virens*	DD (up to 50)	Dose- and time-dependent effects on reproductive and cycle-life	Not detected	[99]
*C. intestinalis*	DD (0.8–8.9)	Delay or block of metamorphosis and decrease of endogenous NO levels	Upregulation of *gclm* and *ggt*	[100]
*C. intestinalis*	DD (2–3.3)	Dose-dependent malformations and delay of larvae	Upregulation of *gclm, gst;* downregulation of *hox1, hox12, cdx*	[101]
*Microzooplankton*	Mixtures of HD (0.005–0.02) and OD (0.0005–0.002)	Dose-dependent delay of growth	Not detected	[102]
*O. dioica*	DD (0.33–16.42), *S. marinoi* and *C. affinis* extracts (5–100)	Dose-dependent aberrations of chordate embryos	Upregulation of *gclm, Aldh3, Aldh2, Aldh8*	[103]
*R. canadum*	DD (0, 0.1, 0.3, 0.5, 0.7, 1.0)	Decreasing of larval survival and juvenile growth	Not detected	[104]

**Table 4:** Biotechnological applications of oxylipins, reporting oxylipins or diatoms analysed, target cells and/or organism and the activity detected. Abbreviations: DD, 2*E*,4*E*-decadienal; DT, 2*E*,4E/*Z*,7*Z*-decatrienal; HD, 2*E*,4*E*-heptadienal; OD, 2*E*,4*E*-octadienal.

Oxylipins/Diatom	Target Cells/Organism	Activity	Reference
DD and DT	Human colon adenocarcinoma (Caco2)	Anticancer	[7]
DD	*B. proboscidea* and *T. heterouncinata*	Anti-parasitic	[119]
DD	*S. salar*	Anti-parasitic	[120]
*C. scutellum parva*	Breast carcinoma (BT20)	Anticancer	[121]
DD, HD, OD	Adenocarcinoma (A549 and COLO 205)	Anticancer	[63]
*C. debaryana*	TNF-α, IL-1β, IL-6 and IL-17	Anti-inflammatory	[122]
*S. marinoi*	Human melanoma (A2058) and *S. aureus*	Anti-cancer and anti-bacterial	[123]
*S. costatum, C. pseudocurvisetus*	*M. tuberculosis* and *M. bovis*	Anti-tuberculosis	[124]
